# Application of Prenatal Whole Exome Sequencing for Congenital Heart Anomalies

**DOI:** 10.3390/ijms27041720

**Published:** 2026-02-10

**Authors:** Threebhorn Kamlungkuea, Fuanglada Tongprasert, Duangrurdee Wattanasirichaigoon, Sirinart Kumfu, Siriporn C. Chattipakorn, Nipon Chattipakorn, Theera Tongsong

**Affiliations:** 1 Fetal Center, Faculty of Medicine, Chiang Mai University, Chiang Mai 50200, Thailand; 2 Department of Obstetrics and Gynecology, Chiang Mai University, Chiang Mai 50200, Thailand; 3 Division of Medical Genetics, Department of Pediatrics, Faculty of Medicine, Ramathibodi Hospital, Mahidol University, Bangkok 10400, Thailand; 4 Cardiac Electrophysiology Research and Training Center, Faculty of Medicine, Chiang Mai University, Chiang Mai 50200, Thailand; 5 Center of Excellence in Cardiac Electrophysiology Research, Faculty of Medicine, Chiang Mai University, Chiang Mai 50200, Thailand; 6 Cardiac Electrophysiology Unit, Department of Physiology, Faculty of Medicine, Chiang Mai University, Chiang Mai 50200, Thailand; 7 Department of Oral Biology and Diagnostic Sciences, Faculty of Dentistry, Chiang Mai University, Chiang Mai 50200, Thailand

**Keywords:** congenital heart disease, prenatal whole exome sequencing, proband, trio-WES, additional diagnostic yield, incremental diagnostic yield

## Abstract

Congenital heart disease (CHD) is the most common congenital anomaly worldwide and poses significant diagnostic challenges due to its structural complexity and frequent association with extracardiac anomalies and genetic abnormalities. While conventional tests such as karyotyping, quantitative fluorescent polymerase chain reaction (QF-PCR), and chromosomal microarray analysis (CMA) are standard first-tier investigations, many cases remain genetically unexplained. Prenatal whole exome sequencing (WES) has emerged as a valuable tool to detect pathogenic single gene variants underlying CHD. This narrative review synthesizes findings from 28 studies involving over 2000 WES-tested fetuses and more than 10,000 CHD cases. The additional diagnostic yield of WES over CMA ranged from 8.0% to 66.7%, with higher yields in syndromic or non-isolated CHD (10–50%) compared to isolated cases (7.1–27.8%). Trio-based WES outperformed proband-only sequencing by improving accuracy, reducing turnaround time, and lowering the rate of variant of uncertain significance (VUS). Prenatal WES not only clarifies genetic etiology but also reveals syndromic diagnoses, allowing CHD to be interpreted within broader multisystem contexts. Integration of phenotypic and genomic data enhances prenatal counseling, prognostication, delivery planning, and postnatal care—advancing precision medicine in fetal cardiology.

## 1. Introduction

Congenital heart disease (CHD) is the most common congenital anomaly worldwide [1]. It caused 261,247 deaths globally in 2017 [2]. The reported incidence of CHD varies across studies, ranging from approximately 4 to 50 per 1000 live births [3]. The overall prevalence has progressively increased to about 1 in 100 live births, or approximately 1% of the population [1,3]. Among these cases, critical CHD accounts for approximately 25–30% of all CHD cases, and often requires intensive postnatal care and early intervention, and may be associated with extracardiac anomalies and neurodevelopmental delays [4,5,6]. However, prenatal detection rates for major CHD vary widely, ranging from 30% to 85%, depending on the quality of screening and the level of sonographer expertise, and only about 56% of major CHD cases are detected prenatally [7,8].

The etiology of CHD is multifactorial, involving a complex interaction of genetic, environmental, and epigenetic factors. Prenatal risk factors for CHD arise from both maternal and fetal conditions. Maternal risk factors include a family history of CHD, coexisting maternal diseases such as diabetes mellitus, collagen vascular disorders, and phenylketonuria, as well as maternal obesity and exposure to teratogens (e.g., lithium, isotretinoin, alcohol, and cocaine). Additional risk factors include chorionic twinning and pregnancies conceived via in vitro fertilization (IVF) [9,10,11]. On the fetal side, the risk is often associated with genetic abnormalities, which account for approximately 30% of all CHD cases, including chromosomal anomalies, copy number variations (CNVs), and single gene mutations [12,13].

The incidence of genetic abnormalities in CHD varies across studies, depending on the genetic work-up protocols and technological capabilities of each institute around the world. Conventional karyotyping remains the most fundamental and widely available laboratory test. The detection rate of aneuploidy and chromosomal abnormalities using karyotyping is approximately 23% [14,15]. For abnormal CNVs in the presence of normal karyotypes, which occur in approximately 10–15% of CHD cases, chromosomal microarray (CMA) can be used to detect these abnormalities. CMA is currently the first-tier prenatal genetic test for congenital anomalies, as recommended by the American College of Obstetricians and Gynecologists (ACOG) and the Society for Maternal–Fetal Medicine (SMFM) [15,16,17]. Additionally, other molecular techniques such as quantitative fluorescence polymerase chain reaction (QF-PCR) and fluorescence in situ hybridization (FISH) are available for aneuploidy and CNVs detection. However, conventional karyotyping, CMA, QF-PCR, and FISH can typically diagnose genetically associated CHD in approximately 30–40% of cases. These tests are unable to diagnose single gene disorders and rare variants, which have been previously reported in approximately 15–40% of CHD cases [18,19]. Beyond identifying the genetic etiology, the detection of pathogenic gene variants can also provide prognostic information. These findings may be associated with syndromes or comorbidities that are not visible on prenatal ultrasound, such as endocrine abnormalities, hypotonia or hypertonia, and neurodevelopmental disorders.

Based on classical embryology, normal organ development and function are regulated by the genetic information encoded within the 46 chromosomes of each cell. When chromosomal abnormalities occur, such as aneuploidy, structural rearrangements, micro- and macro-duplications or deletions, and CNVs, they can disrupt embryogenesis and organ physiology, often resulting in congenital anomalies. Additionally, in some cases, abnormalities arise at a smaller scale, at the level of individual genes, leading to single gene disorders. Common mechanisms of single gene disorders include point mutations, frameshift mutations, and small insertions or deletions. For prenatal diagnosis, beyond standard genetic testing, novel techniques are needed to bridge the existing diagnostic gap. Recently, an advanced molecular genetic test, specifically prenatal whole exome sequencing (WES), has been increasingly studied and reported as a promising tool to address this limitation.

Several systematic reviews and meta-analyses have demonstrated the utility of pre-natal WES with additional diagnostic yields as high as 17.4% [19], as well as a high rate of variant of uncertain significance (VUS) and variants of secondary findings unrelated to the prenatal ultrasound phenotype or associated with late-onset conditions. These findings could complicate the interpretation of clinical significance and increase challenges in parental counseling; therefore, prenatal WES is not currently recommended for routine clinical use and remains largely confined to research settings [20].

This narrative review aims to explore the emerging role of prenatal WES in the evaluation of CHD, with the focus on the following issues: (1) the overall process of prenatal WES from the detection of prenatal ultrasound abnormalities to the identification of causative genetic variants; (2) the genetic regulation of cardiac development and its association to CHD; (3) the diagnostic yield of prenatal WES in CHD; (4) the added value of trio-based exome sequencing (sequencing of the fetal and both parental specimens) compared to proband-only sequencing (only the index case or affected fetus) in improving diagnostic rate and accuracy; and (5) the clinical outcomes and prognoses of CHD associated with pathogenic or likely pathogenic (P/LP) genetic variants. Ultimately, a deeper understanding of the whole process of phenotype and genetic diagnosis could elaborate the additional utility and value of prenatal WES in CHD, enhance awareness of clinical outcomes and prognostic implications linked to genetic variants identified, promote its investigative use in uncovering the genetic etiology of CHD, and contribute to more precise prenatal decision-making and clinical management.

## 2. Identification of the Targeted Articles

This comprehensive review was conducted using the PubMed database, covering publications from January 1997 to December 2024. The search keywords included terms such as “prenatal/antenatal/fetal/fetus,” “whole exome sequencing,” “congenital,” “cardiac/heart,” “defect/disease/abnormalities/anomaly,” “gene,” and “development/embryology.” The search yielded a total of 68 relevant original articles related to the genetic regulation of heart development and associated CHD, and 28 articles that specifically addressed prenatal WES in CHD. All selected articles were subsequently incorporated into this review.

## 3. The Process of Prenatal WES: From Prenatal Ultrasound to Causative Genetic Variant

**Figure 1:**
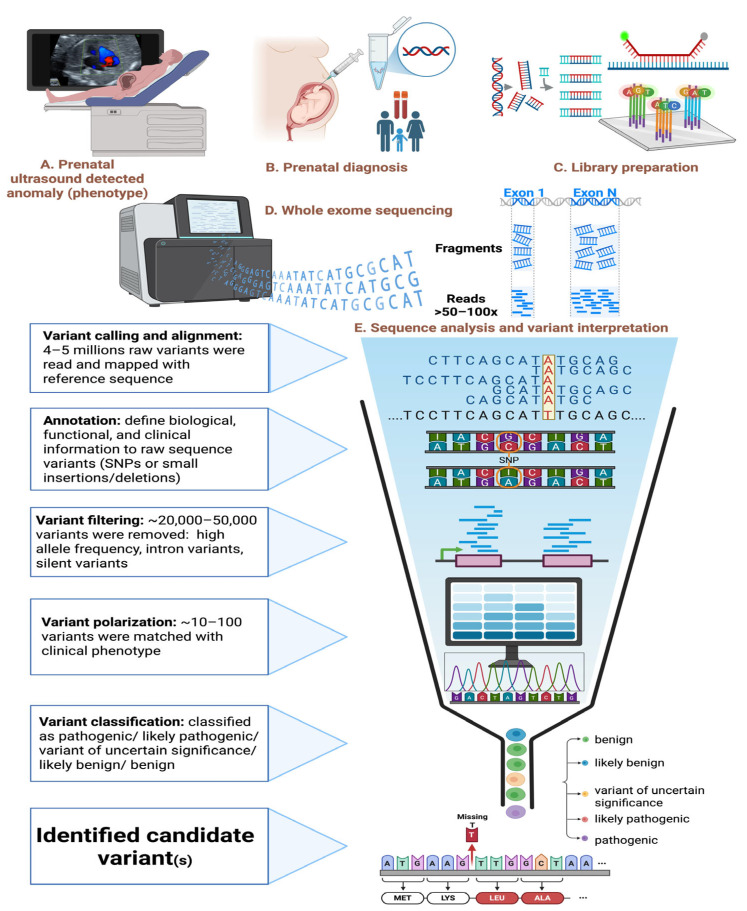
Schematic overview of the prenatal diagnostic workflow. (**A**) Prenatal ultrasound identifies a fetal anomaly (e.g., congenital heart disease). (**B**) Prenatal diagnosis is pursued, involving extraction of fetal (and parental) DNA. (**C**) Library preparation is performed, including DNA extraction, DNA fragmentation, end repair and A-tailing, adapter ligation, PCR amplification, and exome capture hybridization. (**D**) Whole exome sequencing is carried out on the prepared library. (**E**) Sequence analysis and variant interpretation are performed using a bioinformatics pipeline, encompassing variant calling and alignment, annotation, variant filtering, variant prioritization, and variant classification, to identify the causative variant(s). Abbreviation: SNPs, single nucleotide polymorphisms. The figure was created with BioRender.com (Ruankham, P., 2026; retrieved from https://BioRender.com/empele5 (accessed on 29 January 2026)).

Prenatal WES is a genetic diagnostic technique used to identify pathogenic variants in the protein-coding regions (exons) of the fetal genome, particularly in cases of congenital anomalies, syndromic conditions, or suspected monogenic disorders. After obtaining a prenatal specimen (e.g., chorionic villi, amniotic fluid, or fetal blood), the WES process involves three main laboratory steps: library preparation, generation of the DNA sequence (sequencing), and data analysis (Figure 1) [21].

In prenatal WES for congenital heart anomalies, genomic DNA extracted from the prenatal sample undergoes exome enrichment using hybridization with biotin-labeled probes targeting exonic regions, which comprise only 1–2% of the genome but often represent about 85% of disease-related variants. The enriched exonic DNA is captured with streptavidin-coated magnetic beads, then amplified and sequenced to generate millions of short DNA fragments with detailed nucleotide information (A, T, C, G) [22]. After sequencing, the data analysis involves a comprehensive bioinformatics pipeline, including quality control, sequence assembly, mapping to a reference genome (e.g., GRCh38), variant calling, annotation, filtering, prioritization, and clinical interpretation. Raw data are stored in FASTQ files, aligned reads in BAM files, and variants are identified using tools such as GATK, producing VCF files that catalog differences like SNVs, indels, and structural variants. From an initial pool of 4–5 million variants, WES typically captures approximately 20,000–25,000 raw exonic variants per individual. Variants are annotated with biological and clinical context, including gene information, mutation types, predicted functional effects, population frequencies, and references from databases such as ClinVar and OMIM, along with pathogenicity scores (e.g., SIFT, PolyPhen). Filtering reduces the list to potentially pathogenic variants based on quality, frequency (excluding variants with minor allele frequency > 1%), and functional impact—retaining variants such as non-synonymous, frameshift, or splice-site mutations. Prioritization emphasizes variants relevant to the clinical phenotype, known CHD-related genes, inheritance patterns, and computational predictions. This approach facilitates the detection of de novo, compound heterozygous, and X-linked variants, with references from databases such as Online Mendelian Inheritance in Man (OMIM) and Human Phenotype Ontology (HPO). Finally, candidate variants are correlated with the clinical phenotype and classified according to standard guidelines (e.g., ACMG criteria). Variants classified as pathogenic or likely pathogenic (P/LP) are reported as clinically significant findings [23,24,25].

## 4. Genetic Regulation of Heart Development and Associated CHD

The heart is the first organ to form in the embryo. The development of the heart comprises five main mechanisms: (1) formation of the cardiogenic plates and heart tube; (2) rotation and folding of the heart tube; (3) chamber formation and patterning; (4) vascular and outflow tract development; and (5) development of cardiac conduction system.

### 4.1. Formation of the Cardiogenic Plates and Heart Tube

**Figure 2:**
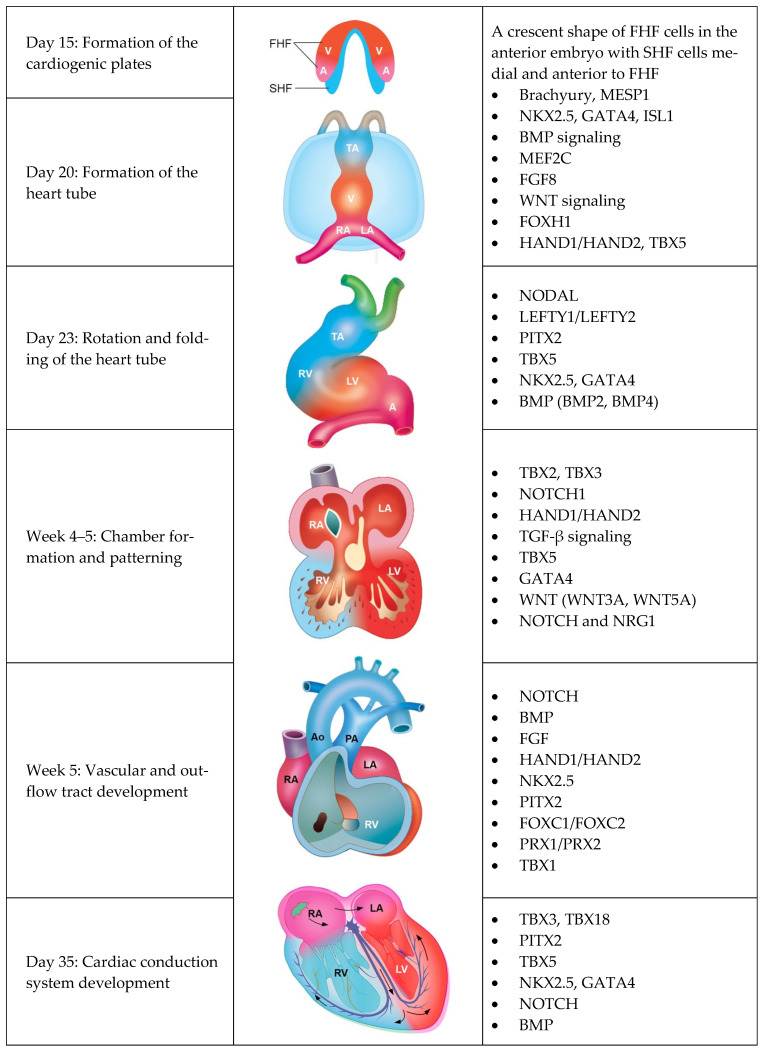
Cardiac development and key genes of genetic control. Abbreviation: A, atrium; Ao, aorta; FHF, first heart field; LA, left atrium; LV, left ventricle; PA, pulmonary artery; RA, right atrium; RV, right ventricle; SHF, second heart field; TA, truncus arteriosus; V, ventricle.

At the third week after conception, the embryo consists of three germ layers: ectoderm, mesoderm, and endoderm. In the splanchnic layer of the lateral plate mesoderm, clusters of angiogenic cardiac progenitor cells migrated bilaterally and longitudinally along the foregut. Starting on day 18 after conception, cardiogenic mesenchymal cells originate on both sides of the embryonic midline, giving rise to the first heart field (FHF) on the anterior lateral side and the second heart field (SHF) on the anterior medial side. The FHF is destined to develop into the heart tube, left ventricle, and parts of both atria, while the SHF cardiac precursors migrate first to the pharyngeal region and differentiate into endocardium, myocardium and epicardium contributes to the formation of the right ventricle, additional portions of the atria, and the outflow tract (Figure 2). On the 21st day of development, as the embryo undergoes lateral and cranial folding, the two plates (FHF and SHF) come closer to each other and eventually fuse at the midline, forming a primordial heart tube that will further undergo looping, septation, and chamber formation [26,27,28].

The pivotal genes and transcription factors that regulate cardiac crescent differentiation, specification, and morphogenesis include *ISL1*, *MESP1*, *NKX2.5*, *GATA4*, and *TBX5* (see Table 1 for key genes and regulatory pathways). Abnormalities in the genetic pathways that govern myocyte formation can lead to CHD such as double-outlet right ventricle (DORV), pulmonic stenosis, and tetralogy of Fallot (TOF), as well as right-sided disorders including hypoplastic right ventricle, Ebstein’s anomaly, and arrhythmogenic right ventricular dysplasia [26,29,30].

### 4.2. Rotation and Folding of the Heart Tube

By the fourth week, the dorsal mesocardium, which attaches the primitive heart tube to the surrounding structures, dissolves. The heart tube then undergoes segmental enlargement into five portions, listed from superior to inferior as follows:

- **Truncus arteriosus:** arising from the bulbus cordis and later developing into the ascending aorta and pulmonary trunk.
- **Bulbus cordis:** consisting of the conus cordis and the lower part of the ventricle, which will eventually form the smooth portions of the right and left ventricles.
- **Primitive ventricle:** connected to the primitive atrium via the narrow atrioventricular canal. The region connecting to the bulbus cordis, known as the bulbo-ventricular sulcus, later forms the interventricular groove. The primitive ventricle develops into the trabeculated portions of the ventricles.
- **Primitive atrium:** giving rise to the trabeculated parts of the atria.
- **Sinus venosus:** a thin-walled, sac-like structure formed by the confluence of the left and right sinus horns. It later contributes to the smooth part of the right atrium, the coronary sinus, and the vein of the left atrium.

On the 23rd day of embryonic development, the bulbus cordis undergoes rotation in an inferior, anterior, and rightward direction, while the primitive ventricle rotates superiorly and leftward, resulting in the ventricle becoming ventral to the atrium. This process, known as bulbo-ventricular looping, positions the primitive atrium upward and posteriorly, with the sinus venosus eventually aligning posterior to the primitive atrium. On the 28th day of development, the ventriculo-bulbar portion begins to contract, generating pulsations. Cardiac neural crest cells participate in the formation of the heart outflow tracts. Defects in neural crest derivatives of the heart can be caused by 22q11 deletion (DiGeorge syndrome), which leads to haploinsufficiency of the transcription factor T-Box1 (TBX1), resulting in conotruncal defects phenotype [31,32]. The other associated gene of rotation and folding of the heart tube, such as *TBX5*, *NODAL*, *LEFTY1/LEFTY2*, *PITX2*, and *BMP*, whose mutation results in heterotaxy syndrome, dextrocardia, atrioventricular discordance, and transposition of the great arteries (TGA; Table 1) [33,34,35].

### 4.3. Chamber Formation and Patterning

Between the 4th and 5th weeks, the heart develops its four chambers. The atrioventricular canal is narrow, and the inner walls grow to form a thick septum called the endocardial cushion, which separates the canal into left and right sides. This directs blood flow through the two atrioventricular canals. By the end of the 4th week, the septum primum begins to grow downward from the atrium, helping to divide the atria. During this process, an opening called the foramen primum forms, allowing blood flow between the atria. As the septum continues to develop, the foramen primum eventually closes, while small perforations form in the septum primum to create a new opening called the foramen secundum, ensuring continued blood flow during development.

**Table 1:** Key genes and pathway regulation of heart development.

Process	Key Genes	Regulation Pathways	Associated CHDs
Formation of the Cardiogenic Plates and Heart Tube	TBX5 [36], MESP1 [37,38,39,40] NKX2.5, GATA4, ISL1 [41,42,43,44,45] BMP [46,47] MEF2C [48,49,50,51] FGF8 [52,53,54] WNT [55,56,57,58,59] FOXH1 [60]	→ Master regulator initiating cardiovascular progenitor commitment → Essential for heart progenitor differentiation and regional fate determination → Induces cardiac mesoderm and promotes myocardial differentiation → Early cardiac specification → Critical for SHF proliferation and alignment of the outflow tract → Temporally regulated, with WNT inhibition promoting cardiac differentiation → Essential for development of the SHF	DORV Pulmonic stenosis TOF RV hypoplasia Ebstein’s anomaly Arrhythmogenic RV dysplasia
2. Rotation and Folding of the Heart Tube	NODAL [61,62,63] LEFTY1/LEFTY2 [62] PITX2 [64,65,66,67,68] TBX1 [32,33], TBX5 [69,70,71,72] NKX2.5, GATA4 [41,51,73] BMP2, BMP4 [35,46,47]	→ Drives the left-right axis patterning → Antagonize NODAL to refine asymmetry → Ensures proper looping direction and chamber alignment → Crucial for atrioventricular and ventricular septation during looping → Essential for myocardial differentiation and structural integrity during looping → Promotes myocardial proliferation and looping directionality	Heterotaxy syndrome, dextrocardia AV discordance Conotruncal defects TGA
3. Chamber Formation and Patterning	TBX2, TBX3 [74,75] NOTCH1 [76] HAND1/HAND2 [77,78] TBX2, TGF-β signaling [79] TBX5 [69,70,71,72] GATA4 [80] WNT3A, WNT5A [55,56,57,58,59,81] NOTCH and NRG1 [76,82,83,84]	→ Induce atrioventricular myocardial development and endocardial cushion formation → Regulates endocardial cushion formation → Early cardiac chamber-specific transcription factors, defining left/right ventricular identity → Modulates epithelial-to-mesenchymal transition (EMT) in endocardial cells → Crucial for atrioventricular septation → Regulates myocardial compaction and septal growth → Regulates polarity and myocardial patterning → Control myocardial migration, chamber formation ventricular trabecular initiation	ASD AVSD Hypoplastic left/right heart syndrome VSD
4. Vascular and Outflow Tract Development	NOTCH [85] BMP FGF [86] Hand1/Hand2 NKX2.5 [87] PITX2 [88,89] FOXC1/FOXC2 [90] PRX1/PRX2 [88] TBX1 [91]	→ Modulate outflow tract development and aortic arch artery patterning → Crucial for outflow tract septation → Contributes to outflow tract septation and valve elongation → Coordinate valve morphogenesis and outflow tract remodeling → Essential for normal outflow tract and right ventricle development → Directs asymmetric remodeling of the outflow tract and aortic arch derivatives → Essential for aortic arch artery patterning and vascular remodeling → Contributes to aortic arch development → Contributes to outflow tract and aortic arch development	TOF Truncus arteriosus DORV IAA Pulmonary artery stenosis Bicuspid aortic valve
5. Cardiac Conduction System Development	TBX3 [92,93,94], TBX18 [95] PITX2 [96] TBX5 [97] NKX2.5 [98,99], GATA4 [100] NOTCH [101] BMP [102]	→ Suppress working myocardium gene expression to establish the SAN → Specifies left-right asymmetry of the SAN → Essential for AVN specification and conduction pathway formation → Regulate AVN and His bundle differentiation → Directs cardiac conduction system lineage commitment and AV conduction system patterning → Promotes pacemaker cell fate in the SAN	AV node hypoplasia Atrial fibrillation Congenital heart block Cardiomyopathy

Abbreviation: ASD, atrial septal defect; AV, atrioventricular; AVN, atrioventricular node; AVSD, atrioventricular septal defect; DORV, double outlet right ventricle; IAA, interrupted aortic arch; RV, right ventricular; SAN, sinoatrial node; SHF, second heart field; TGA, transposition of the great artery; TOF, tetralogy of Fallot; VSD, ventricular septal defect.

After the septum primum forms, the septum secundum develops on the right side and partially fuses with the endocardial cushion, leaving an opening called the foramen ovale. Blood flows through the foramen ovale to the left atrium, with the septum primum acting like a valve to regulate this flow. Atrial septation is regulated by a network of transcription factors (NOTCH1, GATA4, TBX5), signaling molecules (BMPs, WNTs), and structural proteins. Disruption in these genes can lead to atrial septal defects (ASD), particularly ostium secundum ASD and atrioventricular septal defect (AVSD) [35,69].

By the end of week 4, the muscular interventricular septum begins growing from the midline toward the endocardial cushion, dividing the ventricles into left and right chambers. Initially, the septum does not fully fuse, leaving the interventricular foramen, an opening that allows communication between the ventricles. This opening is later closed when the bulbar ridge and the inferior endocardial cushion grow downward and fuse with the muscular septum, forming the membranous interventricular septum. This process relies on signals along the left-right axis, regulated by key transcription factors like HAND1 (mainly involved in left ventricular development) and HAND2 (mainly involved in right ventricle formation), collectively known as Heart and Neural Crest Derivatives Expressed 1 and 2. Mutations or haploinsufficiency in these genes can lead to congenital heart defects such as hypoplastic left or right heart syndrome and ventricular septal defects (VSD) [28,103]. Other essential genes are also involved (Table 1).

### 4.4. Vascular and Outflow Tract Development

In the fifth week, the truncus arteriosus and the upper part of the bulbus cordis (conus cordis) are separated by the growth and fusion of ridges on opposite sides, forming the spiral (aorticopulmonary) septum. This divides the truncus into the pulmonary artery and aorta, and forms the outflow tracts of the ventricles.

At the same time, tissue around the atrioventricular orifice grows inward to form the atrioventricular valves. The chordae tendineae develop from this tissue and connect to papillary muscles inside the ventricles. Additionally, during weeks 5 to 7, mesenchymal tissue in the conotruncal region creates ridges that develop into the three cusps of the semilunar (aortic and pulmonary) valves. The key genes regulating vascular and outflow tract development include *NOTCH*, *BMP*, *HAND1/HAND2*, *NKX2.5*, *PITX2*, and *FOXC1/FOXC2*. Alterations or abnormalities in these genes can disrupt the developmental mechanisms regulating outflow tract formation, leading to congenital heart malformations such as TOF, persistent truncus arteriosus, DORV, interrupted aortic arch, pulmonary artery stenosis, and bicuspid aortic valve (Table 1) [85,104].

### 4.5. Development of Cardiac Conduction System

During cardiac chamber specification, the cardiac conduction system develops concurrently. The rhythmic contraction of the atria and ventricles is regulated by the coordinated function of two primary electrical nodes: the sinoatrial node (SAN) and the atrioventricular node (AVN). Around day 35, myocardial precursor cells differentiate into specialized conduction cells. The SAN, which develops from tissue in the sinus venosus or on the ventrolateral surface of the superior vena cava, acquires autonomous electrical activity and serves as the primary pacemaker of the heart. It is anatomically located in the sulcus terminalis on the inner wall of the right atrium. The development and regulation of these cells are primarily controlled by *TBX5* and *TBX18* [97,105].

Shortly after SAN formation, electrical impulses begin to propagate through the AVN (also known as the Aschoff-Tawara node). The upper portion of the AVN originates from the sinus venosus, while the lower portion arises from the atrial canal. The AVN is in the myocardium at the base of the atrioventricular septum. From the AVN, the bundle of His emerges and extends toward the apex of the heart, subsequently dividing into right and left bundle branches. The left bundle branch develops slightly earlier and travels along the interventricular septum. Both branches give rise to Purkinje fibers, which are distributed beneath the endocardium to facilitate synchronized ventricular contraction. The formation and differentiation of the AVN and Purkinje fibers are regulated by *NKX2.5*, and inactivation of this gene has been shown to result in progressive degeneration of the AVN and atrioventricular block [106].

Additionally, pacemaker cell precursors in the sinus node are closely related to the myocardium surrounding the pulmonary veins. The posterior wall of the left atrium extends to and ensheathes the proximal pulmonary veins, establishing electrical continuity. Several studies have demonstrated that atrial fibrillation (AF) often originates from arrhythmogenic foci within the pulmonary veins, and that AF can be effectively treated by electrical isolation of these veins [26,107]. The development of the pulmonary vein myocardium is regulated by the PITX2 transcription factor. Recent genetic studies have identified risk haplotypes at chromosome 4q25, which involve the *PITX2* gene and are associated with increased susceptibility to AF [107,108,109]. Other genes involved in the development of the cardiac conduction system are summarized in Table 1.

## 5. The Additional Diagnostic Yield of Prenatal WES in CHD

Multiple studies have evaluated the additional diagnostic yield of prenatal WES in fetuses with CHD, particularly following negative results from traditional standard genetic tests. The approach to prenatal genetic testing varies among studies and can generally be categorized into two main strategies: the stepwise approach and parallel genetic testing. The stepwise approach is typically preferred in clinical settings by ruling out chromosomal abnormalities and CNVs through initial testing (e.g., QF-PCR, karyotyping, CMA) before proceeding to WES. In contrast, parallel testing, where WES is conducted simultaneously with standard tests, has been primarily demonstrated in research contexts.

Among the reviewed studies employing the stepwise approach, six distinct testing pathways were identified:

- Normal QF-PCR, followed by normal karyotyping and CMA, then WES
- Normal QF-PCR and CMA, followed by WES
- Normal karyotyping, followed by CMA and then WES
- Normal results from either QF-PCR, karyotyping, or CMA, followed by WES
- Normal CMA, followed by WES
- Normal CNV sequencing, followed by WES

Notably, the detection rate of pathogenic or likely pathogenic variants by WES was not significantly influenced by the specific stepwise pathway used.

Across 28 reviewed studies encompassing over 10,000 fetuses with cardiac anomalies and more than 2000 cases undergoing prenatal WES, the additional diagnostic yield of WES ranged from 8.0% to 66.7%. This variation largely depended on factors such as study design, the type of WES performed (e.g., proband-only vs. trio-based WES), the specific subtype of CHD, and whether the CHD was isolated or associated with extracardiac anomalies. Studies that employed trio-based WES (e.g., Lord et al. (2019), Yi et al. (2022), Li et al. (2023), Normand et al. (2018), Koning et al. (2019)) generally demonstrated higher diagnostic sensitivity compared to those using proband-only sequencing, reflecting the added value of parental data for interpreting variant inheritance and de novo status [25,105,110,111,112]. A summary of WES diagnostic yields across studies, including breakdowns by study cohort, genetic testing approach, WES strategy, and CHD type (isolated vs. non-isolated), is presented in Table 2.

Among the 28 studies evaluating prenatal WES in CHD, only study by Qiao et al. (2021) reported an additional diagnostic yield of less than 10%, specifically 8% [116]. The remaining studies demonstrated yields greater than 10%, with more than half reporting additional diagnostic yields exceeding 20%. This lower detection rate can be explained by the study by Qiao et al. (2021), which included a large distribution of different CHD phenotypes (360 unselected fetuses), particularly a large proportion of isolated CHD (77%), which can affect the overall detection rate [113]. The highest detection rates were reported by Koning et al. (2019) at 66.7% [25], followed by Leung et al. (2018) at 42.9% [131], and Lai et al. (2022) at 34.2% [130].

Isolated CHD cases generally demonstrated lower diagnostic yields from WES, rang-ing from 7.1% to 27.8%, whereas syndromic or non-isolated cases typically exhibited higher yields between 10.5% and 50.0%. Notably, a few studies reported exceptionally high diagnostic yields exceeding 80.0–100% in select subgroups. In some studies, the difference in detection rates between isolated and non-isolated CHD cases was modest. For instance, Lu et al. (2022) reported yields of 11.4% in isolated CHD and 12.5% in non-isolated CHD, while Lin et al. (2024) found similar results with 12.2% and 14.3%, respectively [113,125]. However, other studies demonstrated substantial disparities between the two groups. Fu et al. (2018) reported a detection rate of 83.3% in non-isolated CHD compared to just 7.1% in isolated cases [118]. Similarly, Diderich et al. (2021) observed a 100% diagnostic yield in non-isolated CHD, in contrast to 6.3% in isolated cases [129]. The presence of extracardiac features (e.g., limb defects, renal anomalies, facial dysmorphism, CNS findings) can help narrow the differential diagnosis and increase the accuracy of variant interpretation by increasing the phenotypic match in sequencing interpretation and clarifying the VUS. In addition, isolated CHD is more likely to be multifactorial, in-volving subtle gene–gene or gene–environment interactions not detectable by WES alone. The isolated structural defects without other anomalies may also arise from non-coding variants, epigenetic changes, or hemodynamic influences, which WES may not capture [18,126].

Moreover, several studies have specifically examined the diagnostic yield of prenatal WES in distinct subtypes of CHD, revealing variability in detection rates among different anatomical and phenotypic categories. For example, Yi et al. (2022) reported an additional diagnostic yield of 13% in fetuses with heterotaxy [111]. Sun et al. (2020) discovered a yield of 27.8% in cases of noncompaction cardiomyopathy [132]. Li et al. (2023) reported a 28.6% yield in fetuses presenting with a single atrium or single ventricle [112]. Sacco et al. (2024) found a diagnostic yield of 37.5% in isolated conotruncal anomalies and an even higher yield of 45.5% in conotruncal anomalies associated with syndromic features [133].

## 6. The Utility of Trio-Based Exome over Proband-Only Sequencing in Improving Diagnostic Accuracy

Although prenatal WES has proven to be a powerful tool with substantial diagnostic yield following negative results from standard genetic testing, several limitations and concerns remain regarding its application in daily clinical practice. These include the frequent identification of VUS, long turnaround times, the possibility of incidental or secondary findings, and challenges related to cost and insurance coverage [20,134].

VUS are a common challenge in WES and are particularly difficult to interpret in the absence of a comprehensive phenotypic context. According to systematic reviews and meta-analyses, the pooled incremental rate of VUS ranges from 15.5% to 26% [18,19], which may lead to unclear genetic counseling, increased parental anxiety, and complex clinical decision-making. However, the use of trio-based exome sequencing, which includes analysis of the fetus (proband) alongside both parents, can help mitigate these challenges. As summarized in Table 3, trio-based WES not only improves diagnostic yield but also reduces the rate of VUS and enhances variant interpretation, owing to the availability of parental genotypes that allow for more accurate classification of inheritance patterns and variant pathogenicity.

Among studies using predominantly trio-based WES (e.g., Koning et al. (2019) [25], Westphal et al. (2019) [121], Marangoni et al. (2022) [21]), diagnostic yields ranged from 13.0% to the highest diagnostic yield of 66.7% in a small cohort, with zero VUS. In contrast, studies employing proband-only WES (e.g., Hu et al. (2018) [114], Tan et al. (2022) [115], Lu et al. (2022) [113]) demonstrated moderate diagnostic yields (11.5–20.6%) but higher VUS rates, reaching up to 28.8% in Lu et al. [113]. These findings highlight a key limitation of proband-only WES, the greater difficulty in interpreting isolated variants without parental genotype data.

**Figure 3:**
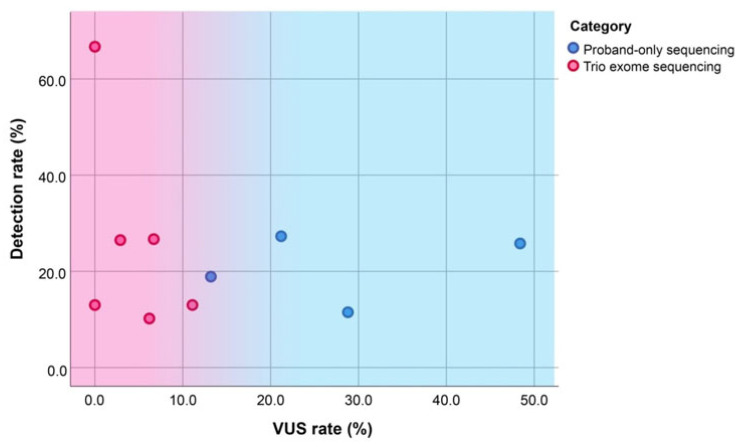
Scatterplots of the reported detection rates against VUS rates according to the WES approaches. Abbreviation: VUS, variant of uncertain significance.

Some studies employed a combined approach, performing proband-only WES in certain cases and trio exome sequencing in others. For example, Fu et al. (2018) reported an additional diagnostic yield of 20.6% with a VUS rate of 11.8%; notably, all VUSs were identified exclusively in cases that underwent proband-only sequencing [118]. Similarly, Chahwan et al. (2022) demonstrated an intermediate diagnostic yield of 25.8% but reported a notably high VUS rate of 48.4%, likely attributed to the limited use of trio-based analysis [120]. The comparison of additional diagnostic yield and the detection rate of VUS in proband-only sequencing, combined approach (in which some cases used proband-only and others used trio-based analysis), and trio-based sequencing, is presented in Figure 3.

In the context of prenatal care, turnaround time (TAT) remains a critical limitation of WES. The TAT for prenatal WES varies depending on the specific protocols and sequencing platforms used by individual laboratories, typically ranging from 2 to 8 weeks or long-er [135,136]. This timeframe may be significantly delayed informing time-sensitive prenatal decision-making, particularly in cases where legal or ethical limits on pregnancy termination often apply before 20 to 24 weeks of gestation, depending on national regulations. The TAT also varies significantly depending on the sequencing strategy employed. Several studies have reported faster results with trio-based WES, particularly when prioritized for clinical decision-making. For example, Normand et al. (2018) documented a TAT of approximately 2 weeks for trio-based WES, compared to over 12 weeks for proband-only sequencing [110]. Across studies utilizing proband-only approaches, including those by Hu et al. (2018) [114], Fu et al. (2018) [118], Chahwan et al. (2022) [120], and Normand et al. (2018) [110], the average TAT ranged from approximately 3 to 12 weeks. In contrast, studies that primarily employed trio-based sequencing strategies, such as those by Koning et al. (2019) [25], Dempsey et al. (2021) [119], Marangoni et al. (2022) [21], and Li et al. (2020) [122], reported substantially shorter TATs, ranging from less than 17 days to 8 weeks. These findings suggest that trio-based WES may offer not only higher diagnostic utility but also timelier results in the prenatal setting.

In addition, trio-based exome sequencing allows for the determination of parental origin, mode of inheritance, and zygosity of the detected gene variant, such as whether it is maternally, paternally inherited, or de novo. This information provides significant utility for future pregnancy planning, recurrence risk assessment, and personalized prenatal management. These findings underscore the value of trio-based WES in the prenatal setting, not only for improving diagnostic yield in CHD but also for reducing uncertainty and facilitating more confident prenatal counseling.

## 7. Prognosis and Outcomes of CHD Cases Associated with Pathogenic/Likely Pathogenic Variants

Across the reviewed studies, as summarized in Table 4, the prognosis and pregnancy outcomes of fetuses with CHD carrying P/LP variants varied widely. Management decisions were largely influenced by the severity of the cardiac defect, study protocols, and the ethical and legal frameworks specific to each country. For instance, Sun et al. (2020) reported on fetuses with noncompaction cardiomyopathy, in which 33 cases (89%) with P/LP variants were electively terminated following ultrasound diagnosis and informative counseling [132]

In several studies, termination was permitted even at later gestational ages, and in such contexts, all cases with P/LP variants resulted in elective termination. For example, Li et al. (2023) [112] diagnosed CHD between 17 and 22 weeks of gestation, Xue et al. (2024) [22] between 13 and 27 weeks, and Lin et al. (2024) [125] between 20 and 28 weeks; in all these studies, pregnancies with confirmed P/LP variants were terminated. These findings underscore the significant impact of timely genetic results and local policies on prenatal decision-making. 

For cases in which the pregnancy was continued despite the presence of P/LP variants associated with CHD, outcomes varied. Some were complicated by stillbirth or pre-term birth, while others resulted in livebirths with palliative care or early neonatal death. For example, in the study by Dempsey et al. (2021), 3 out of 7 fetuses with P/LP variants continued to term, and one case proceeded with postnatal palliative care [119]. Similar outcomes were reported in studies by Marangoni et al. (2022) [21] and Lai et al. (2022) [130]. In the study by Marangoni et al. (2022), among 9 P/LP cases, 6 pregnancies were terminated, 2 resulted in stillbirth, and 1 was a liveborn neonate who died on day 2 of life [21].

Similarly, Lai et al. demonstrated that 10 of 13 pregnancies with P/LP variants were terminated, 2 developed stillbirths, and 1 resulted in a preterm birth with neonatal death on the day of delivery [130]. These findings suggest that CHD cases associated with P/LP variants are often associated with poor perinatal outcomes, including high rates of pregnancy termination, stillbirth, and early neonatal death.

Moreover, fetal phenotyping during pregnancy is often limited, as certain abnormalities such as craniofacial dysmorphisms, central nervous system malformations, neuro-muscular dysfunction, and neurodevelopmental deficits may be difficult or impossible to detect prenatally. As a result, some fetuses diagnosed with CHD and caring P/LP variants were later found to have unrecognized extracardiac anomalies at birth, which adversely affected growth and postnatal surgical outcomes. For example, Koning et al. (2019) re-ported 2 live births among fetuses with CHD and P/LP variants [25]. One neonate died shortly after birth due to airway obstruction, while the other experienced preterm birth and died on 18 days of life following cardiac surgery [25]. Similarly, Diderich et al. (2021) de-scribed 6 liveborn cases of CHD with P/LP variants, all of whom were later identified to have extracardiac anomalies, including agenesis of the corpus callosum, craniofacial malformations, or limb abnormalities [129]. These findings highlight the limitations of prenatal imaging and the importance of integrating genetic data to anticipate broader syndromic outcomes.

Many genetic variants associated with CHD are linked to syndromes involving multiple organ anomalies and neurodevelopmental impairments. For example, Diderich et al. (2021) reported a fetus initially presenting with AVSD; prenatal WES revealed a *PTPN11* mutation, consistent with a diagnosis of Noonan syndrome [129]. In the same study, another fetus with complex cardiac anomalies was found to have a *MASP1* mutation, indicative of 3MC syndrome, a condition characterized postnatally by developmental delay, intellectual disability, hearing loss, and growth restriction, all of which are typically undetectable prenatally [129]. Similarly, Hu et al. (2018) reported a fetus with isolated TOF whose prenatal WES identified a *CHD7* mutation, confirming CHARGE syndrome, of which the associated chorioretinal coloboma, cranial nerve dysfunction, ear malformations, and significant postnatal growth and developmental delays are often missed on prenatal imaging [114].

These cases illustrated the concealed diagnostic value of prenatal WES. In scenarios where prenatal ultrasound identifies isolated cardiac anomalies or with non-lethal extracardiac findings and standard genetic testing returns negative results, WES can uncover underlying syndromic conditions that may not be clinically diagnosed during pregnancy. The identification of a pathogenic variant not only clarifies the genetic etiology but also facilitates targeted evaluation for additional anomalies, enhances prognostic accuracy, and informs perinatal and postnatal management strategies. Thus, the utility of prenatal WES extends beyond the detection of genetic causes of CHD alone; it serves as a powerful tool in revealing broader syndromic diagnoses that might otherwise remain unrecognized until after birth. Ultimately, prenatal WES provides comprehensive information that empowers healthcare providers to inform families to make precise, timely decisions that are in the best interest of both the fetus and the family.

## 8. Conclusions

Prenatal WES offers substantial additional diagnostic value in fetuses with CHD, particularly following negative or inconclusive results from standard genetic testing. The overall additional diagnostic yield of WES in CHD ranges from 8.0% to 66.7%, with a lower yield between 7.1% and 27.8%, and significantly higher yield ranging from 10.5% to 50% in non-isolated or syndromic CHD, and even 80.0–100% in selected studies. The rate of VUS varies from 10% to 30%, depending on the sequencing approach. Trio-based WES has demonstrated superior performance, offering higher diagnostic yield, shorter turnaround times, and a lower VUS rate, often below 10%, while also enabling determination of variant inheritance patterns. Beyond establishing the genetic etiology of CHD, prenatal WES can reveal associated extracardiac anomalies, improve prognostic accuracy, and guide individualized perinatal and postnatal management strategies tailored to specific gene mutations. The comprehensive insights provided by WES support precise clinical decision-making by facilitating the targeted selection of CHD cases for advanced genetic evaluation. Ultimately, this approach enhances diagnostic efficiency and optimizes care planning, thereby providing significant benefits to both the fetus and the family.

## 9. Future Research Perspectives

This review of gene regulation and prenatal WES highlights the substantial diagnostic value and clinical utility of WES in the evaluation of CHD. To advance its broader clinical application, future research should focus on assessing the cost-effectiveness and practical utility of different genetic testing strategies, particularly stepwise versus parallel approaches. A key strength is the comparison between trio-based WES and proband-only sequencing, with trio analyses consistently demonstrating higher diagnostic yields and lower rates of VUS. This clarity in stratification offers new insights into how prior testing practices influence incremental diagnostic yield. Moving forward, the development of a prospective, standardized reporting framework would facilitate meta-analytic synthesis, emphasizing that trio-based prenatal WES provides meaningful diagnostic improvements and reduces uncertainty—thereby offering clearer guidance for counseling and perinatal planning. Integrating artificial intelligence (AI) holds promising potential in this area. AI could enhance prenatal ultrasound interpretation by improving the accuracy of phenotypic recognition and anomaly detection, thereby enabling more targeted genetic testing. Furthermore, AI-driven bioinformatics tools may streamline sequencing analysis, reducing turnaround time while increasing the precision of variant classification and gene-disease association. Due to the time-sensitive nature of prenatal decision-making and gestational age limits, reliance on stepwise testing may become increasingly impractical. Instead, comprehensive genomic approaches may evolve toward WGS, which captures both coding (exonic) and non-coding regions. WGS enables simultaneous analysis of single nucleotide variants, copy number variants, and structural variants, including inversions, translocations, and insertions, offering a more complete genetic landscape for fetal evaluation. This shift could ultimately transform prenatal diagnostics, enabling earlier and more accurate decision-making for families and clinicians.

## Figures and Tables

**Table 2:** Additional detection yield of pathogenic/likely pathogenic variant in congenital heart disease by prenatal WES.

Study	Prenatal Study Cohort	Number of Cases	Genetic Approach	WES Approach	CHD with Prenatal WES	P/LP Variant Cases	Detection Rate (%)
					Total Cases (Isolated/Non-Isolated)	Total Cases (Isolated/Non-Isolated)	Total (Isolated/Non-Isolated)
Lu, 2022 [113]	Cardiac anomalies	200	Normal CMA ⟶ WES	Proband	52 (44/8)	6 (5/1)	11.5 (11.4/12.5)
Hu, 2018 [114]	Cardiac anomalies	1126	Normal karyotyping ⟶ Normal CMA ⟶ WES	Proband	44 (33/11)	7 (3/4)	15.9 (9.1/36.4)
Tan, 2022 [115]	Cardiac anomalies	121	Combined low-coverage WGS and WES	Proband	53 (23/30)	10 (4/6)	18.9 (17.4/20.0)
Qiao, 2021 [116]	Cardiac anomalies	360	Normal karyotyping/CMA ⟶ WES	Combined	300 (243/57)	24 (18/6)	8.0 (7.4/10.5)
Sun, 2020 [117]	Left-sided heart defect	80	Normal CNV sequencing ⟶ WES	Combined	66 (53/13)	13 (8/5)	19.7 (15.1/38.5)
Fu, 2018 [118]	Congenital anomalies	3988	Normal QF-PCR ⟶ Normal karyotyping ⟶ Normal CMA ⟶ WES	Combined	34 (28/6)	7 (2/5)	20.6 (7.1/83.3)
Dempsey, 2021 [119]	Congenital anomalies	52	Normal CMA ⟶ WES	Combined	32 (NA/NA)	7 (NA/NA)	21.9 (NA/NA)
Chahwan, 2022 [120]	Congenital anomalies	122	Normal karyotyping/CMA ⟶ WES	Combined	31 (12/19)	8 (1/7)	25.8 (8.3/36.8)
Marangoni, 2022 [21]	Congenital anomalies	303	Normal QF-PCR ⟶ Normal CMA ⟶ WES	Combined	34 (NA/NA)	9 (NA/NA)	26.5 (NA/NA)
Westphal, 2019 [121]	Cardiac anomalies	30	Normal karyotyping/CMA/SGS ⟶ WES	Combined	30 (15/15)	8 (3/5)	26.7 (20.0/33.3)
Normand, 2018 [110]	Congenital anomalies	146	Normal karyotyping/CMA ⟶ WES	Combined	37 (NA/NA)	11 (NA/NA)	29.8 (NA/NA)
Li, 2020 [122]	Cardiac anomalies	260	Normal karyotyping/CMA ⟶ WES	Trio	260 (190/70)	26 (16/10)	10.0 (8.4/14.3)
Mone, 2021 [123]	Cardiac anomalies	147	Normal QF-PCR ⟶ Normal karyotyping/CMA ⟶ WES	Trio	107 (85/22)	11 (8/3)	10.3 (9.4/13.6)
Yi, 2023 [124]	Cardiac anomalies	736	Normal CNV sequencing ⟶ WES	Trio	301 (206/95)	32 (18/14)	10.6 (0/14.7)
Lord, 2019 [105]	Congenital anomalies	744	Normal QF-PCR ⟶ Normal CMA ⟶ WES	Trio	193 (122/71)	24 (14/10)	12.4 (11.5/14.1)
Lin, 2024 [125]	Cardiac anomalies	1118	Normal CMA ⟶ WES	Trio	62 (41/21)	8 (5/3)	12.9 (12.2/14.3)
van Nisselrooij, 2020 [126]	Cardiac anomalies	727	Normal CMA ⟶ WES	Trio	108 (NA/NA)	14 (NA/NA)	13.0 (NA/NA)
Xing, 2022 [127]	Cardiac anomalies	586	Normal QF-PCR/Karyotyping/CMA ⟶ WES	Trio	47 (19/28)	7 (2/5)	14.9 (10.5/17.9)
Yates, 2017 [128]	Terminated anomalous	84	Normal karyotyping/CMA ⟶ WES	Trio	26 (NA/NA)	6 (0/6)	23.1 (NA/NA)
Diderich, 2021 [129]	Cardiac anomalies	391	Normal CMA ⟶ WES	Trio	44 (32/12)	14 (2/12)	31.8 (6.3/100)
Lai, 2022 [130]	Congenital anomalies	93	Normal QF-PCR/Karyotyping/CMA ⟶ WES	Trio	38 (NA/NA)	13 (NA/NA)	34.2 (NA/NA)
Leung, 2018 [131]	Congenital anomalies	33	Normal QF-PCR/Karyotyping/CMA ⟶ WES	Trio	7 (NA/NA)	3 (1/2)	42.9 (NA/NA)
Koning, 2019 [25]	Congenital anomalies	22	Normal karyotyping/CMA ⟶ WES	Trio	6 (2/4)	4 (0/4)	66.7 (0/80.0)
Yi, 2022 [111]	Heterotaxy	135	Normal CNV sequencing ⟶ WES	Trio	69 (0/69)	9 (0/9)	13.0 (0/13.0)
Xue, 2024 [22]	Dextrocardia	29	Normal karyotyping/CMA ⟶ WES	Trio	15 (11/4)	3 (1/2)	20.0 (9.1/50.0)
Sun, 2020 [132]	NCCM	37	Normal CNV sequencing ⟶ WES	Trio	20 (18/2)	5 (5/0)	25.0 (27.8/0)
Li, 2023 [112]	Single atrium/ventricle	44	Normal karyotyping/CMA ⟶ WES	Trio	7 (4/3)	2 (1/1)	28.6 (25.0/33.3)
Sacco, 2024 [133]	Conotruncal anomalies	302	Normal QF-PCR/CMA ⟶ WES	Trio	16 (5/11)	6 (1/5)	37.5 (20.0/45.5)

Abbreviation: CHD, congenital heart disease; CMA, chromosomal microarray; CNV, copy number variants; NA, not available; NCCM, noncompaction cardiomyopathy; P/LP, pathogenic/likely pathogenic; QF-PCR, quantitative fluorescent polymerase chain reaction; SGS, single gene sequencing; WES, whole exome sequencing; WGS, whole genome sequencing.

**Table 3:** Efficacy of prenatal WES: Proband-only vs. Trio-based sequencing.

Study	WES Approach	Prenatal Specimen	Number of Cases	Number of P/LP Variants	Number of VUS	Detection Rate (%)	VUS (%)	Turnaround Time
Hu, 2018 [114]	Proband	CVS, AF, cord blood	34	7	4	20.6	11.8	3 wk
Tan, 2022 [115]	Proband	AF, cord blood, heart tissue	53	10	7	18.9	13.2	NA
Lu, 2022 [113]	Proband	AF	52	6	15	11.5	28.8	NA
Fu, 2018 [118]	75.0% Proband 25.0% Trio	CVS, AF, cord blood	34	7	4 (Proband-only)	20.6	11.8	3 wk
Chahwan, 2022 [120]	91.0% Proband 9.0% Trio	CVS, AF, cord blood	31	8	15	25.8	48.4	12 wk
Normand, 2018 [110]	Trio and Proband	CVS, AF, cord blood	37	11	NA	29.7	NA	Proband: 12.6 wk Trio: 2 wk
Dempsey, 2021 [119]	74.4% Trio	CVS, AF	32	7	0	21.9	0	14–17 d
Sun, 2020 [117]	78.8% Trio	Cord blood	66	13	5	19.7	7.6	NA
Westphal, 2019 [121]	83.3% Trio	CVS, AF, cord blood, skin or umbilical tissue	30	8	2	26.7	6.7	3–12 wk
Marangoni, 2022 [21]	96.3% Trio	CVS, AF, cord blood	34	9	1	26.5	2.9	17–43 d
Koning, 2019 [25]	Trio	CVS, AF	6	4	0	66.7	0	<17 d
Li, 2020 [122]	Trio	CVS, AF	260	26	16	10.0	6.2	3–8 wk
Mone, 2021 [123]	Trio	CVS, AF	107	11	5	10.3	4.7	NA
Yi, 2022 [111]	Trio	Cord blood	69	9	0	13.0	0	NA
van Nisselrooij, 2020 [126]	Trio	Prenatal diagnosis procedures	108	14	12	13.0	11.1	NA

Abbreviation: AF, amniotic fluid; CVS, chorionic villous sampling; d, days; NA, not available; P/LP, pathogenic/likely pathogenic; VUS, variant of uncertain significance; WES, whole exome sequencing; wk, weeks.

**Table 4:** Prognosis and outcomes of CHD cases associated with genetic variants.

Study	GA at CHD Diagnosed	Turnaround Time	Prenatal WES Cases	P/LP Cases	Pregnancy Outcome of P/LP Variants (Cases)	Neonatal Outcome
Normand, 2018 [110]	NA	2 wk	37	11	TOP (all)	-
Sun, 2020 [132]	20–33 wk	NA	20	5	TOP (all)	-
Li, 2023 [112]	17–22 wk	NA	7	2	TOP (all)	-
Xue, 2024 [22]	13–27 wk	NA	15	3	TOP (all)	-
Lin, 2024 [125]	20–28 wk	NA	62	8	TOP (all)	-
Hu, 2018 [114]	24–27 wk	3 wk	44	7	TOP (4), continued pregnancy (3)	NA
Mone, 2021 [123]	17–26 wk	NA	107	11	TOP (6), stillbirth (1), livebirth (4)	NA
Westphal, 2019 [121]	12–36 wk	3–8 wk	30	8	TOP (6), livebirth (2)	NA
Dempsey, 2021 [119]	NA	NA	32	7	TOP (4), livebirth (3)	1 case: livebirth and received palliative care after birth 2 cases: livebirth and remain under pediatric follow-up
Marangoni, 2022 [21]	NA	2–6 wk	34	9	TOP (6), stillbirth (2), livebirth (1)	1 case: neonatal death at day 2
Lai, 2022 [130]	NA	4 wk	38	13	TOP (10), stillbirth (2), preterm birth (1)	1 case: preterm birth with neonatal death at day 0
Koning, 2019 [25]	Before 22 wk	7–19 d	7	4	TOP (2), livebirth (2)	1 case: neonatal death from airway obstruction in the NICU 1 case: preterm birth with neonatal death at day 18 post-surgery
Li, 2020 [122]	11–35 wk	3–8 wk	260	26	TOP (18), neonatal death (2), livebirth (4), loss to follow-up (2)	2 cases: neonatal death 2 cases: loss to follow-up
Diderich, 2021 [129]	Before 24 wk	NA	44	14	TOP (7), livebirth (6), loss to follow-up (1)	All cases: livebirth and had extra-cardiac anomalies, e.g., corpus callosum agenesis, craniofacial or limb abnormalities
Xing, 2022 [127]	Before 28 wk	NA	47	6	TOP (5), livebirth (1)	1 case: livebirth and underwent postnatal cardiac surgery with no extra-cardiac anomalies identified

Abbreviation: CHD, congenital heart disease; d, days; GA, gestational age; P/LP, pathogenic/likely pathogenic; NA; not available; TOP, termination of pregnancy; wk, weeks; WES, whole exome sequencing.

## Data Availability

The datasets analyzed during the current study are available from the corresponding author upon reasonable request.
